# Stimulants in Fever

**Published:** 1846-12

**Authors:** 


*Stimulants in Fever.* By Dr. Corrigan, Dublin.—"Let us, as we stand at the bed-side of a patient in typhus fever, recollect that in looking at the extent of the maculæ, or for the presence of petechiæ,or examining the distended vessels of the conjunctivæ, we are looking not at a mere local derangement, but that we are studying in these external indications the state generally of the circulatory system as a whole. In this view we can understand why we attach importance to the color of the maculæ, why we look upon rose-colored maculæ as a good sign, and dark-colored maculæ as indications of danger.

The dark-colored maculæ are indications of danger, because their color is owing, we know, to an enfeebled circulation. The feebler it is, the darker will be the color of the maculæ; while the more energetic is the capillary circulation, the more vivid will be color of the blood passing through it. In this view we can also find an explanation of the fact, that a patient may have an intellect not disturbed, may have a cool skin, a clean tongue, a soft abdomen, a pulse not above 70 or 80, with volition and sensibility perfect—and yet die of typhus fever in seven or eight days. Of what does the patient die in such a case? He dies of this lesion of the function of circulation. In most cases this lesion is not only the one present, although often the most prominent; but I wish to fix attention on it in this that I may call an analytic view of fever, as it leads to practical rule for the administration of one of our most important agents in the treatment of fever, viz., wine. You are too often bewildered in the directions as to its employment. You are told to beware of delirium in its administration, and yet, again, you read that delirium subsides under its use. You read instructions either to refrain from its use when the tongue is dry, or to judge of the propriety of continuing its exhibition by its effects on the tongue. Instead of attempting to reconcile all the contradictory statements, and, too often, inexplicable advices, that are laid down for you, turn from the books to the living book—the patient—and read from him. Ask yourself what is it in typhus fever you prescribe wine for? Is it for delirium? No. Is it to prevent its approach? Again, no. Do you give it for a dry tongue? Certainly not. What is it that, as you consider a patient's state, would lead you to think of its employment?—is it not the state of the function of circulation, taken as a whole, indexed to you by the pulse, on one hand, and by the state of the capillary system of circulation in the skin, on the other.

It is for this you give it. It is the specific remedy directed to remedy the general lesion of the function of circulation, and hence in its administration you may give it, and you must give it, whether there is or whether there is not delirium; for delirium may be present or absent in a case requiring its exhibition for the function of circulation. You should give it indifferently, whether the tongue is moist or dry, for the tongue may be either, and yet wine may be required; and hence the tongue becoming moist is not an indication that you may dispense with its use—nor its continuing dry, a sign to make you discontinue it. You may give it with a soft abdomen, or with an abdomen tympanitic, for similar reasons. You are giving wine, recollect, as the specific remedy for the lesion of the function of circulation (remember always comprising under this the capillary and cardiac circulation); and by the change in the circulation, and by this alone, you are to judge of the neccessity of continuing, decreasing, or augmenting its dose. Under its exhibition, you will see the vessels of the conjunctiva contract, the maculæ become rose-colored, and the patches of skin in the face, and on dependent portions of the body, lose their dark livid hue. Keep this, then, in mind, the lesion in fever for which you give wine, is the lesion of circulation, and if this function from debility require it, you must give it under all circumstances of derangement of other funtions. Of the quantity required, it is quite impossible to lay down any rule. No two cases will have exactly the same amount of depression of circulatory energy; no two cases will require precisely the same amount of wine. In some cases, four to six ounces are enough for a few days, in continuance, to restore the circulation to sufficient tone; in other cases it requires as much as one ounce of wine every hour, ar 24 ounces in 24 hours; and even in addition to this, as much as eight ounces of brandy; and all this barely sufficient to preserve the circulation from sinking.

We must never abandon a case of fever, as long as there is life; we must remember what the post mortem of the case of Rodmond tells us, that in its present form there is no structural disease; that the patient, even in the dying moment, is sinking from a mere lesion of function, and that even then, recovery is not hopeless; and we must recollect what clinical observation of several cases even now under our own observation in the hospital tells us, that the patient one day, in a state seemingly moribund, may on next day, or within twenty-four hours, be out of danger. It will not unfrequently happen, that even the power of swallowing is lost for several hours; that a small portion of brandy or wine can only be got down by raising the patient in bed, throwing spoonfuls of brandy into the pharynx, and then holding up the patient's head until it descends to the stomach, apparently almost by its gravity. Even thus it sometimes cannot be passed along to the stomach, but even then we can stimulate the circulating system by injections; and in some cases which you have seen, I am confident the preservation of life has been owing to æther, given in the form of injection every two hours, in quantities of two drachms, until under its stimulating effect the circulation gained some vigor, and the power of swallowing returned.

With the same object in stimulating the capillary circulation, blisters are applied in succession over the surface. The nurse is supplied with four or six blisters; one after another is applied, with intervals of six hours between them, over chest, abdomen, thighs, and legs. They are thus applied, not as as counter-irritants, not to act as derivatives on internal structural disease, but as stimulants to excite the capillary system. An action produced in any part of it, will be conveyed through the whole, and thus their action on the skin coincides with and assists the action of the internal stimulus of wine, ammonia, and brandy."—*Dublin Hospital Gazette.*

